# Effect of Paraplegia on the Time Course of Exogenous Fatty Acid Incorporation Into the Plasma Triacylglycerol Pool in the Postprandial State

**DOI:** 10.3389/fphys.2021.626003

**Published:** 2021-02-03

**Authors:** David W. McMillan, Gregory C. Henderson, Mark S. Nash, Kevin A. Jacobs

**Affiliations:** ^1^Department of Physical Medicine and Rehabilitation, University of Miami Miller School of Medicine, Miami, FL, United States; ^2^The Miami Project to Cure Paralysis, University of Miami Miller School of Medicine, Miami, FL, United States; ^3^Department of Nutrition Science, Purdue University, West Lafayette, IN, United States; ^4^Department of Kinesiology and Sport Sciences, University of Miami, Miami, FL, United States

**Keywords:** spinal cord injury, stable isotope lipid tracer, dietary fat metabolism, neurogenic obesity, autonomic nervous system

## Abstract

Spinal cord injury (SCI) results in disordered fat metabolism. Autonomic decentralization might contribute to dyslipidemia in SCI, in part by influencing the uptake of dietary fats through the gut-lymph complex. However, the neurogenic contributions to dietary fat metabolism are unknown in this population. We present a subset of results from an ongoing registered clinical trial (NCT03691532) related to dietary fat absorption. We fed a standardized (20 kcal⋅kgFFM^–1^) liquid meal tolerance test (50% carb, 35% fat, and 15% protein) that contained stable isotope lipid tracer (5 mg⋅kgFFM^–1^ [U-^13^C]palmitate) to persons with and without motor complete thoracic SCI. Blood samples were collected at six postprandial time points over 400 min. Changes in dietary fatty acid incorporated into the triacylglycerol (TAG) pool (“exogenous TAG”) were used as a marker of dietary fat absorption. This biomarker showed that those with paraplegia had a lower amplitude than non-injured participants at Post_240_ (52.4 ± 11.0 vs. 77.5 ± 16.0 μM), although this failed to reach statistical significance (*p* = 0.328). However, group differences in the time course of absorption were notable. The injury level was also strongly correlated with time-to-peak exogenous TAG concentration (*r* = −0.806, *p* = 0.012), with higher injuries resulting in a slower rise in exogenous TAG. This time course documenting exogenous TAG change is the first to show a potential neurogenic alteration in SCI dietary fat absorption.

## Introduction

Spinal cord injury (SCI) results in dysregulation of energy metabolism that increases the risk of cardiometabolic disease (CMD) (Nash et al., 2019). The Consortium for Spinal Cord Medicine’s recent Clinical Practice Guidelines for diagnosis and management of CMD in SCI (Nash et al., 2019) note a high prevalence of obesity and unique clustering of CMD risk factors related to fat metabolism (Libin et al., 2013). Unique dyslipidemia following SCI includes an increased adiposity per unit body mass index (BMI) (Spungen et al., 2000; Clasey and Gater, 2005; Gorgey and Gater, 2011; Gorgey et al., 2011; Yarar-Fisher et al., 2013; Beck et al., 2014; Cirnigliaro et al., 2015), reduced high-density lipoproteins (HDL) (Bauman et al., 1999; Myers et al., 2007; Groah et al., 2009; Wahman et al., 2010), elevated fasted triglyceride (TAG) (Bauman et al., 1999; Myers et al., 2007; Groah et al., 2009; Wahman et al., 2010), and exaggerated postprandial lipemia (Nash et al., 2005; Emmons et al., 2010, 2014; Ellenbroek et al., 2014). Despite the plausible dietary contributions to dyslipidemia in SCI (Bigford and Nash, 2017), little is known about the acute physiological and fat trafficking responses to feeding in SCI persons. Of particular interest are the potential neurogenic contributions in SCI, given the autonomic nervous system’s role in dietary fat absorption. Specifically, the absorption of fat requires the trafficking of dietary fats from the gut to the circulation via mesenteric lymph vessels. Movement of absorbed material through lymphatic vessels is augmented by contraction of smooth muscle cells surrounding lacteals, a process modulated by the autonomic nervous system (Choe et al., 2015; Bachmann et al., 2019). In mice, both parasympathetic and sympathetic synapses are found in the mesenteric and intestinal lacteals, but thoracic duct innervation comes primarily from sympathetic fibers (Bachmann et al., 2019) suggesting a unique role for sympathetic efferents. In humans direct evidence is not available, but sympathetic efferents to the mesenteric lymph likely arise from the celiac and superior mesenteric ganglion (nerve roots T5 – T12) that innervate the small intestine (Standring, 2021) and liver (Yi et al., 2010). Thus SCI above ∼T4 influences autonomic control of the gut, liver, and likely lymphatics in a manner that could influence fat metabolism, as suggested by altered profiles of lipoprotein subfractions observed in persons with injuries above T4 compared to below (La Fountaine et al., 2017). Furthermore, postprandial lymphatic fat trafficking involves lipoprotein remodeling and occurs in an organ-specific manner primarily accounted for by mesenteric and hepatic lymph (Gracia et al., 2020). Combined, these pre-clinical and clinical studies suggests a possible neurogenic component to dietary fat absorption in SCI.

Determination of dietary fat absorption in SCI could be relevant to the pathogenesis of dyslipidemia in SCI. Still, it would also have implications for the neurological contributions to postprandial fat metabolism in general. Thus, the purpose of this study was to determine the time course of the circulating abundance of dietary fats in the plasma TAG pool after feeding of a standardized meal in persons with and without SCI.

## Methods

### Participants

Eight adult males without SCI (CON) and eight with chronic thoracic SCI (PARA) participated. CON and PARA participants had similar height, body mass, and body mass index, but the PARA group had lower whole-body lean mass and higher body fat percentage (Table 1). All PARA participants but one had neurologically complete injuries based on the International Standards for the Neurological Classification of Spinal Cord Injury (ISNCSCI) (Kirshblum et al., 2011). Impairment Scale (AIS; Table 1). The injury level was mostly in the high thoracic region (6 participants with injuries at/above the T6 level). All procedures were approved by the University of Miami IRB (ID: 20180450) and are available on ClinicalTrials.gov (NCT03691532).

**TABLE 1:** Characteristics of study participants.

	CON	PARA	*p*-value
Age (yr)	32.6 ± 9.2	34.1 ± 11.7	0.780
Height (m)	1.80 ± 0.05	1.76 ± 0.06	0.105
Weight (kg)	84.8 ± 17.3	73.7 ± 8.7	0.129
BMI (kg/m^2^)	26.0 ± 4.7	24.0 ± 3.1	0.326
Duration (y)	–	8.5 ± 6.5	–
Level	–	>T6 = 6/<T7 = 2	–
AIS	–	7 = A/1 = B	–
HR_peak_ (b/min)	155.3 ± 9.4	160.4 ± 11.5	0.345
VO_2 peak_ (L/min)	2.38 ± 0.57	1.53 ± 0.56	0.009
VO_2 peak_ (mL/kg/min)	28.7 ± 8.0	21.4 ± 9.0	0.111
Lean (kg FFM)	66.8 ± 8.6	52.1 ± 4.3	0.001
% fat	20.6 ± 6.3	29.0 ± 4.0	0.006

*CON, non-injured; PARA, paraplegia; BMI, body mass index; FFM, fat-free mass, peak heart rate (HRpeak) and rate of whole-body oxygen consumption (VO_2peak_) determined via arm cycling graded exercise test, Level of injury and American Spinal Injury Association Impairment Scale (AIS) determined by International Standards for Neurological Classification of Spinal Cord Injury (ISNCSCI) exam.*

### Experiment

Following informed consent, participants underwent dual-energy x-ray absorptiometry (DXA) to assess three-compartment body composition (Discovery-A, Apex 4.0.2 Software, Hologic Corporation). Cardiorespiratory fitness was determined in both groups during graded arm cycle exercise to volitional exhaustion while assessing the whole-body rate of oxygen consumption (VO_2peak_). On another day, at least 96 h later, subjects returned to the laboratory to complete a postprandial lipemia tracer test. Following arrival at the laboratory between 7:00 and 9:00 AM, the experiment assessed energy expenditure at rest (EE) during 10 min of indirect calorimetry (IDC). Following IDC sampling, participants breathed through the same mask instead of a low-volume (39 ml) 2-way non-rebreathing valve (Series 2600, Hans Rudolph). Expired air was directed by the valve down a pulmonary tube (approximately 1.1 L of volume) into a 4 L mixing chamber. After breathing into this chamber for 5 min, the participant’s mixed expired pulmonary gas was sampled via a 60-ml syringe from the back of the mixing chamber and injected into 10-ml gas sampling tubes (Exetainer, Labco). After breath sampling, an intravenous catheter (20–23 g) was inserted into a dorsal hand vein, and arterialized (Horning et al., 1998) blood was collected into an ETDA-containing vacutainer. All blood samples were immediately centrifuged and then aliquoted and stored at −80°C until analysis. Following baseline breath and blood sampling, participants sat for 1 h of rest. After that, a pre-meal blood sample was collected, and participants consumed ∼0.5 L of a liquid test meal containing 20 kcal/kg FFM and 5 mg/kg FFM of stable isotope [U-^13^C]palmitate (Cambridge Isotope Laboratories, Inc., United States) at a population-specific macronutrient distribution (50:35:15 CHO:LIPID:PRO) equal to *ad libitum* SCI norms (Farkas et al., 2019). Development of this meal has been previously described (McMillan et al., 2019), and the use of [U-^13^C]palmitate was guided by the primary objective in the ongoing trial (NCT03691532). Test meals of this composition have previously been shown in obese persons without SCI to result in an isotopic enrichment (IE) of palmitate of approximately 5.5 mol percent excess (MPE) (Davitt et al., 2013). At six time points up to 400 min, post-meal breath and blood samples were collected in the same manner as the baseline sample.

### Sample and Data Analysis

Lipids were extracted from test meals aliquots and plasma. The TAG pool was isolated by thin-layer chromatography (TLC) from plasma lipid extractions. Total lipid from the meal and the isolated TAG from plasma were analyzed by gas chromatography/mass spectrometry (GC/MS) (Metabolic Solutions, Inc., Nashua, NH, United States) for determination of ^13^C enrichment. Concentrations of circulating TAG were determined in-house on an automated analyzer on a Roche Cobas 6000 analyzer (Roche Diagnostics) using manufacturer’s reagents. Intra- and inter-assay% CVs for TAGs were and 1.6 and 2.1, respectively. The percent contribution of dietary fat to plasma TAG was calculated as IE plasma/IE meal × 100, where IE plasma represents IE of palmitate in plasma TAG, and IE meal represents IE of meal palmitate. This percent and the total TAG data were used to calculate TAG’s absolute concentrations from fatty acids in the test meal (exogenous TAG). IE plasma values were corrected for pre-meal baseline IE.

## Results

**FIGURE 1:**
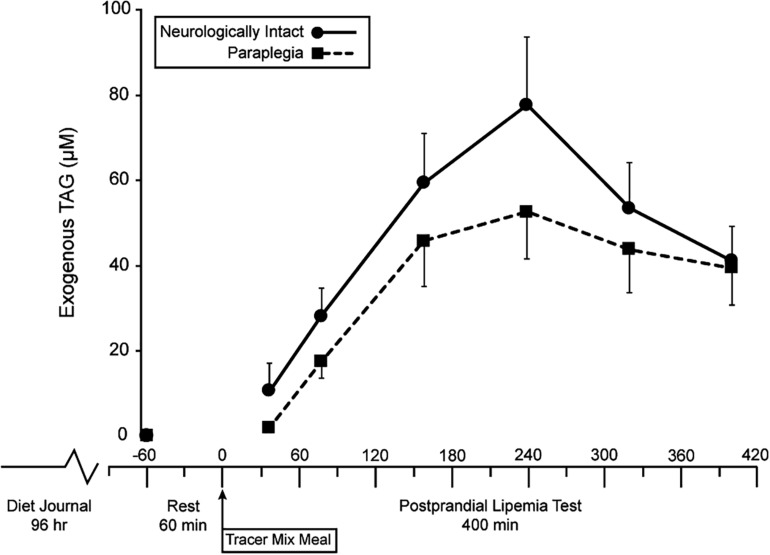
Time course of changes in circulation exogenous triacylglycerides in persons with and without paraplegia. TAG, triacylglycerides, Tracer Mixed Meal = 20 kcal⋅kgFFM^–1^ at 50% carb, 35% fat, 15% with 5 mg⋅kgFFM^–1^ [U-^13^C]palmitate; main effect of time (*p* = 0.002) with no effect of group (*p* = 0.328).

**FIGURE 2:**
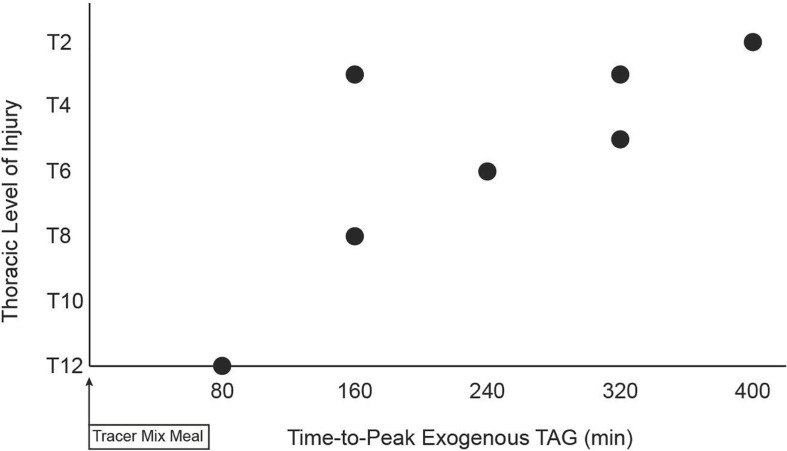
Relationship between level of spinal cord injury and time-to-peak of circulation exogenous triacylglycerides following consumption of a standardized liquid mixed meal. TAG, triacylglycerides, Level of Injury determined by International Standards for Neurological Classification of Spinal Cord Injury (ISNCSCI) exam, Tracer Mixed Meal = 20 kcal⋅kgFFM^–1^ at 50% carb, 35% fat, 15% with 5 mg⋅kgFFM^–1^ [U-^13^C]palmitate; a strong negative correlation (*r* = −0.806) was observed (*p* = 0.012).

The meal size was scaled to each subject’s amount of metabolically active tissue (fat-free mass). Therefore, a lesser lean mass in PARA, compared to CON, resulted in PARA consuming a meal with lower energy (1,041 ± 87 vs. 1,335 ± 171 kcal, *P* < 0.001) and fat content (40.80 ± 3.39 vs. 52.30 ± 6.70, *P* < 0.001). Figure 1 shows the time course of circulating exogenous TAG. Group (PARA vs. CON) by time (7-time points) mixed model analysis of variance found no main effect of group on exogenous TAG (*p* = 0.328). However, an examination of data revealed a difference in exogenous TAG’s time course between the two groups. Notably, CON had little variability in exogenous TAG (7 of 8 participants peaking at 240 min). At the same time, PARA showed wide variability in exogenous TAG values (Figure 2). Further analysis revealed that the time-to-peak exogenous TAG in PARA was mostly explained by the injury level (Figure 2; *r* = 0.802, *p* = 0.012).

## Discussion

Clinical practice guidelines for CMD in this population acknowledge SCI’s pronounced effect on obesity and dyslipidemia (Nash et al., 2019), and impaired postprandial fat metabolism has been described (Nash et al., 2005; Emmons et al., 2010, 2014; Ellenbroek et al., 2014). However, the mechanisms of impaired postprandial fat metabolism have yet to be understood. Using the exogenous TAG biomarker, we report the first evidence in persons with SCI that level of injury influences dietary fat absorption.

Our finding of a strong relationship between the level of injury and time-to-peak exogenous TAG is indirect evidence supporting neurogenic alteration in dietary fat absorption in SCI. Dietary fats in intestinal epithelial cells are packaged into chylomicrons and then transported, via lymphatic vasculature, to the thoracic duct where exogenous TAG enters the circulation and is trafficked toward cellular storage as well as cycling through the VLDL pool and other lipid pools (Knuth and Horowitz, 2006; Heath et al., 2007; Puga et al., 2012). Regarding our methodology, it has been shown that labeled palmitate has low excretion (Jones et al., 1999) and is integrated into circulating chylomicron-TAG (Hodson et al., 2009) in a manner equivalent to or better than other dietary fatty acids. Regarding our population, the autonomic nervous system has been shown to modulate smooth muscle cells’ pulsatile contraction, surrounding lacteals, that drives the efficient movement of material through lymphatic vessels (Choe et al., 2015; Bachmann et al., 2019). The human thoracic duct is innervated by adrenergic nerves (Telinius et al., 2014), and adrenergic agonists increase the contractility of isolated (Telinius et al., 2014) and *in vivo* (Bachmann et al., 2019) lymphatic vessels. Lymphatic innervation suggesting that sympathetic efferents are involved in the modulation of lymphatic contractile transport. Sympathetic efferents exit the spinal column in a segmental fashion making it so in SCI the degree of sympathetic dysfunction is related to the level of injury. For example, persons with an injury below the sixth thoracic vertebra (T6) do not commonly experience the sympathetically driven peripheral cardiovascular event known as autonomic dysreflexia (Karlsson, 1999). It has yet to be determined if sympathetic inputs to gut-associated lymphatic vessels directly modulate the trafficking of dietary fats and thus regulate dietary fat absorption. However, if sympathetic inputs augment the lymphatic trafficking of dietary fats, persons with higher injuries would have a blunted rate of dietary fat absorption. Examination of Figure 2 shows a strong linear relationship despite an outlier with an injury at the T3 level and a 160 min time-to-peak exogenous TAG. If indeed the relationship in Figure 2 is being driven by the effect of SCI on sympathetic-lymphatic connection, then the gradient response suggests that no single neurological level is responsible for the net neurological contribution to absorption of dietary fat. This would suggest that the wide range of thoracic vertebral nerve roots feeding the mesenteric sympathetic efferents reach their end targets via a diffuse, and possibly overlapping, innervation strategy.

Many variables can influence the concentration of TAG derived from fatty acids in the test meal. Our method of introducing the palmitate tracer through the ingestion of labeled dietary fats is advantageous because exogenous TAG can only enter the system through dietary fat absorption. First, slow gastric emptying/motility rates have been reported following SCI (Gondim et al., 2001), although delayed emptying has thus far been shown in persons with cervical level injuries (Zhang et al., 1994; Segal et al., 1995; Kao et al., 1999). Furthermore, liquids’ emptying occurs more quickly than solids (Maurer, 2015), so any effect of SCI in our study would be effecting an emptying time that is relatively short compared to the variability in time-to-peak exogenous TAG. Given the wide range of time-to-peak exogenous TAG in SCI (up to 400 min post-ingestion), variables beyond gastric emptying must be considered (Mackiewicz-Milewska et al., 2016). It is well established that ingested dietary fat is digested into monoacylglycerol (MAG) and FFA within the intestinal lumen, followed by re-esterification of FFA onto MAG in the enterocytes (Phan and Tso, 2001). Our approach of introducing tracer via labeled palmitate takes advantage of this metabolic pathway to label chylomicrons, from which point the labeled dietary fats are integrated into existing lipid pools. Therefore, we track dietary fats’ incorporation into the plasma TAG pool over time with this approach, providing a valuable index of dietary fat absorption. This method does not measure rates of appearance or disappearance of exogenous TAG across the plasma pool but rather provides a time-related profile for the exogenous TAG biomarker level. Therefore, it is possible that differences in the disposal of dietary fat (instead of, or as well as, absorption) influenced the time course of exogenous TAG in SCI. However, some variables known to influence disposal were controlled in our study. Most importantly, SCI reduces fat oxidation due to reduction in lean mass, which differed between the groups (Table 1). However, our meal was standardized to the mass of metabolically active tissue. Furthermore, cardiorespiratory fitness (i.e., peak rate of whole-body oxygen consumption; VO_2peak_), a measure of oxidative capacity, was similar in PARA and CON (when normalized to body mass; Table 1) and in PARA was not related to the level of injury (*p* = 0.101). When considering the principles of the present methodology and the experimental controls that we applied, it appears plausible that dietary fat absorption kinetics have a predominant influence on exogenous TAG biomarker readout.

This brief report shows that the level of SCI influences time-to-peak exogenous TAG, and therefore presents the first indirect evidence of impaired dietary fat absorption in SCI. The use of biomarkers such as exogenous TAG can be used to understand the pathophysiology of impaired fat metabolism in SCI, but when used in this population also allows for an understanding of the role of the central nervous system in regulating dietary fat metabolism.

## Data Availability Statement

The raw data supporting the conclusions of this article will be made available by the authors, without undue reservation.

## Ethics Statement

The studies involving human participants were reviewed and approved by the University of Miami. The patients/participants provided their written informed consent to participate in this study.

## Author Contributions

DM contributed to study design, data collection, data analysis and interpretation, and original draft preparation of the manuscript. GH and MN contributed to data analysis and interpretation and manuscript review and editing. KJ contributed to study design, data analysis and interpretation, and manuscript review and editing. All authors contributed to the article and approved the submitted version.

## Conflict of Interest

The authors declare that the research was conducted in the absence of any commercial or financial relationships that could be construed as a potential conflict of interest.
